# A giant lobular thoracic ganglioneuroma cause skeletal erosion: A case report and literature review

**DOI:** 10.1097/MD.0000000000033891

**Published:** 2023-06-09

**Authors:** Haoxiang Zhuang, Zegang Ruan, Chenyang Xu

**Affiliations:** a Department of Thoracic Surgery, Ganzhou People’s Hospital, Nanchang University, Ganzhou, China.

**Keywords:** ganglioneuroma, lobular, posterior mediastinum, skeletal erosion

## Abstract

Ganglioneuroma (GN) is a rare tumor that originates from neural crest cells and can manifest in any location along the sympathetic chain. It typically exhibits a circular or oval shape and does not invade the surrounding tissue destructively and the enormous lobular appearance and erosion of adjacent skeletal tissues are extremely infrequent among GN.

**Patient concerns:**

A 15-year-old girl presented to our thoracic surgery clinic with a large intrathoracic mass that was incidentally discovered on a chest X-ray. Further imaging with computed tomography and magnetic resonance imaging revealed a lobular profile and an aggressive growth pattern of the tumor, which destroyed the vertebral and rib bones. A tissue sample obtained by needle biopsy was subjected to histopathological analysis, which confirmed the diagnosis of a GN.

**Diagnosis:**

Thoracic (posterior mediastinal) GN and Hashimoto’s thyroiditis.

**Interventions:**

After thoracoscopic exploration, a thoracotomy was performed to excise the mass.

**Outcomes:**

The patient recovered well after surgery, had no major complications, and was discharged without any issues. Further follow-up is necessary to clarify the medium to long-term outcome.

**Lessons:**

Based on existing reports, thoracic GN rarely erodes adjacent bone tissue. By examining previously reported cases, we speculate that the lobular morphology of the tumor may be linked to the more aggressive biological behavior of GN. We also discovered that female patients may be more susceptible to bone erosion. However, further research and additional cases are required to confirm these potential associations.

## 1. Introduction

Ganglioneuroma (GN) is an uncommon, benign tumor that arises from neural crest cells in the sympathetic ganglia or adrenal medulla. Typically, GNs present in the retroperitoneum, posterior mediastinum, or adrenal gland, and occasionally in atypical locations such as intracranial and ureter.^[1–3]^ GNs are typically oval or round in shape and do not destructively erode surrounding structures.^[4]^ In this report, we describe a unique instance of a gigantic lobular GN in the thoracic cavity that caused neighboring rib deformity and erosion of nearby vertebral bodies. The guardians of the patient provided consent for the publication of the report, and both the patient and her mother signed the informed consent form.

## 2. Timeline

Important milestones related to our diagnoses and interventions are exhibit in the Table 1.

**Table 1:** Timeline.

Date	Milestones
2023/3/1	The result of CT suggested neurogenic tumor
2023/3/2	The result of MRI suggested the possibility of ganglioneuroma
2023/3/3	Ultrasound-guided needle biopsy of the mass was performed
2023/3/6	Needle biopsy and immunohistochemistry confirmed the diagnosis of ganglioneuroma
2023/3/8	Surgery day
2023/3/11	Discharged

CT = computerized tomography, MRI = magnetic resonance imaging.

## 3. Case presentation

A 15-year-old girl was found to have a large mass in her right chest during a routine chest X-ray examination. She was admitted to the thoracic surgery department with her mother and had no apparent symptoms or abnormal physical examination results. Her medical history was healthy, with no history of trauma, surgery, or special family history, and a normal menstrual history. Laboratory tests showed elevated levels of FT4, AntiTPO, and TGAB (16.51 pmol/L, 56.33 IU/mL, and 1032.34 IU/mL, respectively). Combining the patient’s thyroid ultrasound results, she was diagnosed with Hashimoto’s thyroiditis. No other abnormalities were found in the rest of the laboratory tests.

Subsequently, a chest computed tomography (CT) scan (including plain and enhanced scans) was performed, which revealed a large low-density lobular mass in the right posterior mediastinum, with bone destruction in the adjacent vertebra and deformed thickening in the right 6th to 8th ribs. After enhancement, the mass showed mild to moderate enhancement. Meanwhile, a ribbon-like shadow was found in the lower lobe of the right lung, with bronchial narrowing, and the thyroid gland was found to be enlarged with uneven density. Based on the CT data, the imaging department suspected that the mass was a neurogenic tumor, but no clear diagnosis could be made. After reviewing the literature, we hypothesized that the diagnosis of ganglioneuroma is more probable. However, it is crucial to differentiate it from neuroblastoma, ganglioneuroblastoma intermixed, and other neurogenic tumors.

**Figure 1.**
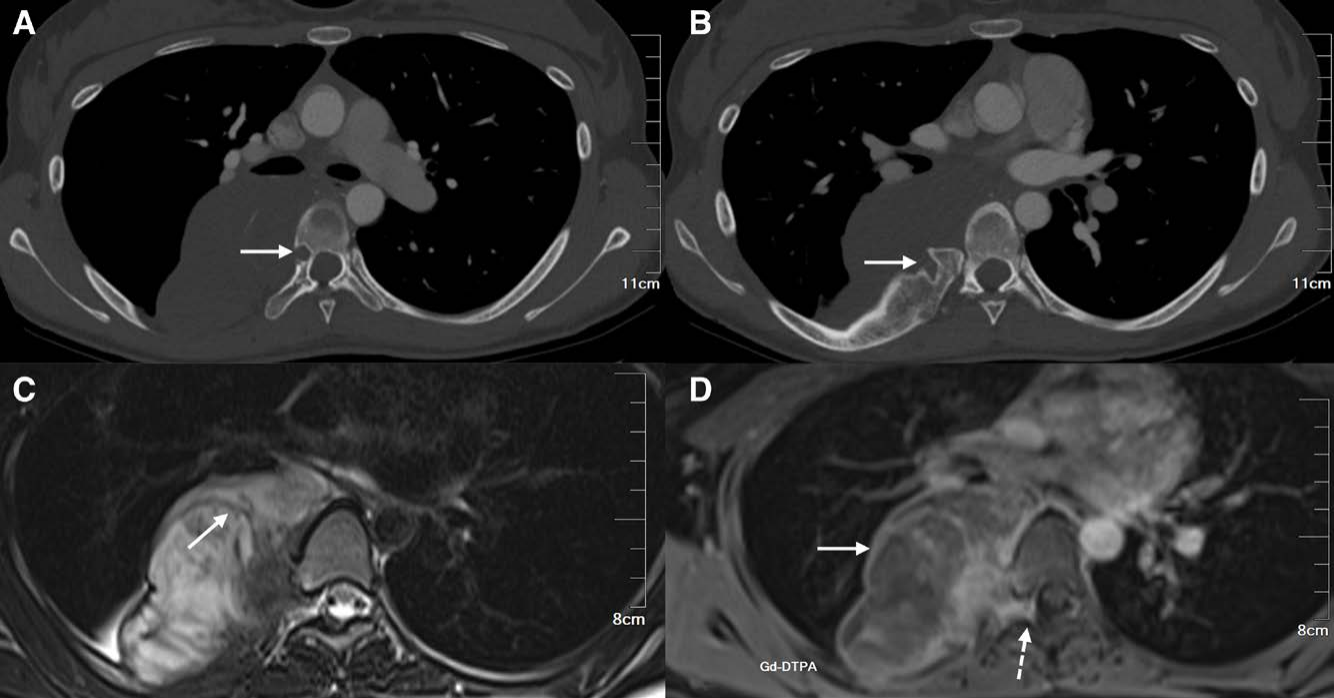
(A): Enhanced CT during arterial phase shows tumor erosion of the right sixth thoracic vertebra, resulting in expansile bone destruction of the vertebral body (white arrow). (B): Enhanced CT during arterial phase shows abnormal proliferation of the right seventh posterior rib with tumor erosion of the bone (white arrow). (C): MRI T2WI shows heterogeneous high signal mixed with linear low signal, presenting as a “whorl” sign (straight arrow). (D): MRI T1 enhanced scan shows more obvious enhancement around the lesion, with tumor extension into the right intervertebral foramen of the sixth and seventh thoracic vertebrae, but not into the spinal canal (dashed arrow). CT = computed tomography, MRI = magnetic resonance imaging, T2WI = T2-weighted imaging.

To further clarify the diagnosis and determine whether the tumor invaded the spinal canal, a thoracic MRI scan was performed. The mass showed slightly low signal intensity on T1-weighted images and slightly high signal intensity with linear low signals on T2-weighted images. After enhancement, most of the lesions showed mild to moderate uneven enhancement with obvious enhancement around the lesions. Based on the MRI findings, the imaging department considered the mass to be a ganglioneuroma (Fig. 1).

A needle biopsy of the mass was performed under ultrasound guidance, which appeared as a low echo with clear boundaries and irregular shape under ultrasound, with uneven internal echo and visible fine strip-shaped blood flow signals on CDFI. The pathology results showed that the mass was a ganglioneuroma, and the immunohistochemistry results showed: S-100(+), SOX-10(+), NF(+), CK(−), SMA(−), Nestin(−), Vim(+), Ki67(1%+), CD34(−), and Desmin(−).

**Figure 2.**
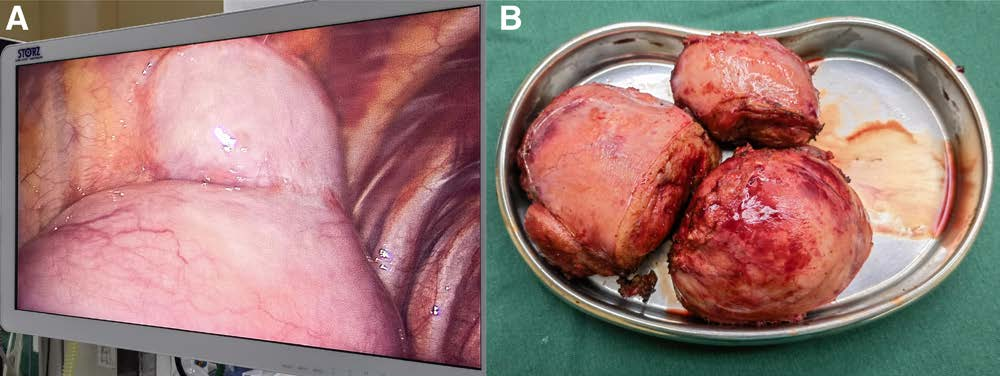
(A): Under thoracoscopy, the tumor presents obvious lobulation with intact capsule. (B): Postoperative tumor specimens consisted of three irregularly shaped tumor tissues with a grayish-red, gray, and white coloration, measuring a total of 14.5 × 12 × 5 centimeters. The cut surface displayed a grayish-white and grayish-yellow appearance, with a soft texture and some areas exhibiting a gelatinous consistency.

**Figure 3.**
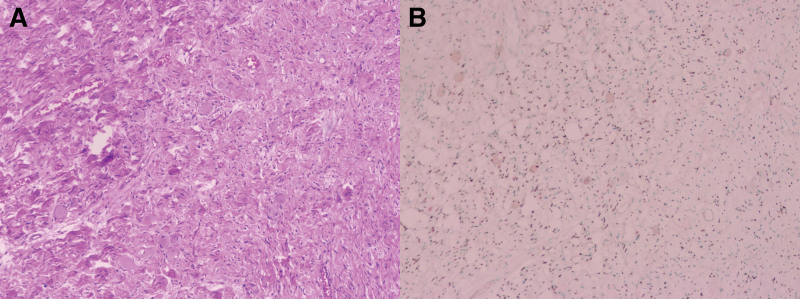
(A): HE staining showing mature ganglion cells and Schwann cells, with original magnification × 100. (B): Ganglion cells display positive staining for SOX-10, with original magnification × 100. HE = hematoxylin and eosin, SOX-10 = SRY-related HMG-box 10.

After excluding surgical contraindications, surgical treatment was performed on the patient. Due to the large size of the tumor, thoracoscopy was first used to explore the lesion and evaluate the feasibility of tumor resection under thoracoscopy. A large lobular mass with intact capsule was seen in the thoracic cavity, due to the large volume of the tumor and its close adhesion to the ribs, right thoracotomy was performed midway through the surgery to remove the tumor. The tumor was divided into three parts for resection (Fig. 2). Considering the risk of complications such as cerebrospinal fluid leakage, The resection was extended to the visible lesion in the intervertebral foramen of the 6th and 7th thoracic vertebrae on the right side. The patient did not experience any significant surgical complications and recovered well. The routine pathological examination of the specimen after surgery was consistent with the results of the needle biopsy (Fig. 3). The patient was smoothly discharged on the tenth day of hospitalization. Currently (3 weeks after operation), there have been no reports of any special discomfort from the patient during telephone follow-up.

## 4. Patient perspective

The patient rejected to share her perspective or experience.

## 5. Discussion

Neuroblastic tumors encompass neuroblastoma, ganglioneuroblastoma (nodular or intermixed), and ganglioneuroma. The International Neuroblastoma Pathology Classification identifies ganglioneuroblastoma intermixed and GN as the mature part of neuroblastic tumors.^[5–7]^ GN is a benign tumor characterized by slow development and lack of invasion. It originates from neural crest cells and is most commonly found in the abdomen/pelvis (66.2%),^[8]^ post mediastinum, and adrenal glands.^[1]^ Thoracic GN usually does not cause symptoms, but in rare cases, it may cause general symptoms such as chest tightness, wheezing, and coughing. If the tumor occurs near the thoracic vertebra, it may cause scoliosis. In cases where the tumor extends to the intervertebral foramina and compresses the spinal cord, it can cause the neurological symptom.^[9,10]^ The majority of intrathoracic GNs present with a regular circular or oval shape and rarely invade surrounding tissues.^[4,11]^ However, in this particular case, the thoracic GN exhibited an extremely rare appearance with massive lobulation and erosion of the adjacent vertebral bodies and ribs, and as the tumor continued to compress and stimulate the surrounding tissues, varying degrees of bone proliferation occurred in the adjacent ribs and vertebrae. Only a few similar cases have been reported in the previous literature.^[12–14]^ In light of these findings, we conducted a review of the literature on intrathoracic GNs from 1990 to the present, to identify commonalities and characteristics of this unusual and destructive growth pattern in intrathoracic GNs.

The inclusion criteria are as follows:

1. Literature related to primary intrathoracic GN.

The exclusion criteria are shown below:

1. Missing critical information about the patient;
2. Secondary intrathoracic GN;
3. Cross-regional GNs, such as extending into the cervical spine or abdomen;
4. GNs in other sites.

We collected a total of 22 articles and 71 cases (including the present case). The clinical data of the patients are shown in Table 2, with a median age of 11 and an average age of 21. The sample included 44 females (62.9%) and 26 males (37.1%), resulting in a female-to-male ratio of 1.7:1. Intrathoracic GN appears to be more common in female patients, which is consistent with previous findings.^8^ Excluding cases with missing data, 6 cases (9.7%) were identified as having lobular GN, while the rest had regular round or oval GN shapes.

**Table 2:** The literature review of primary intrathoracic ganglioneuroma.

Reference	Year	Age (yr)/Gender	Side	Size (cm)	Shape	Skeletal erosion	Rib deformity
Simpson^[12]^	1991	2/F	L	5 × 3 × 2	Regular	No	No
Simpson	1991	5/F	R	10 × 10	Regular	Yes	N/A
Simpson	1991	5/M	R	4 × 3 × 1	Regular	No	No
Simpson	1991	5.5/M	L	8 × 7 × 2	Regular	No	No
Simpson	1991	8/F	L	7 × 5 × 5.5	Regular	No	No
Simpson	1991	9/F	L	N/A	Lobular	Yes	No
Simpson	1991	10/F	L	10 × 7.5 × 7	Regular	Yes	No
Simpson	1991	11F	L	9 × 5 × 3	Lobular	No	No
Simpson	1991	12/F	R	12 × 8 × 2	Regular	No	No
Sakai^[15]^	1992	17/F	L	N/A	Regular	No	No
Osterhouse^[16]^	2002	25/F	L	15 × 7 × 3	Regular	No	Yes
Duffy^[17]^	2005	27/F	R	N/A	N/A	No	No
Velyvis^[10]^	2005	15/F	R	8 × 8 × 2	Regular	No	Yes
Maruyama^[18]^	2007	74/F	R	6.9 × 5.8 × 1.6	Regular	No	No
Ko^[19]^	2007	53/F	R	9 × 4.5 × 10	Regular	No	No
Zhang^[20]^	2009	3/F	L	5.8 × 4.5 × 4.5	Regular	No	No
Kitagawa^[21]^	2010	4/F	R	N/A	Regular	No	No
Guan^[4]^	2012	20/F	N/A	N/A	Regular	No	No
Guan	2012	4/M	N/A	N/A	Regular	No	No
Guan	2012	4/M	N/A	N/A	Regular	No	No
Guan	2012	4/M	N/A	N/A	Regular	No	No
Guan	2012	8/F	N/A	N/A	Regular	No	No
Guan	2012	19/M	N/A	N/A	Regular	No	No
Guan	2012	18/F	R	6.9 × 11.5 × 13	Regular	No	No
Guan	2012	34/M	N/A	N/A	Regular	No	No
Guan	2012	14/F	N/A	N/A	Regular	No	No
Guan	2012	8/M	R	N/A	Regular	No	No
Guan	2012	31/F	N/A	N/A	Regular	No	No
Guan	2012	53/M	R	3.2 × 2 × 2	Regular	No	No
Guan	2012	57/F	N/A	N/A	Regular	No	No
Guan	2012	21/M	N/A	N/A	Regular	No	No
Guan	2012	15/M	N/A	N/A	Regular	No	No
Guan	2012	19/M	N/A	N/A	Regular	No	No
Guan	2012	3/M	N/A	N/A	Regular	No	No
Guan	2012	46/M	N/A	N/A	Regular	No	No
Guan	2012	11/M	N/A	N/A	Regular	No	No
Guan	2012	14/M	L	7 × 5 × 3	Regular	No	No
Guan	2012	47/F	N/A	N/A	Regular	No	No
Guan	2012	22/M	R	6.7 × 4.5 × 2.5	Regular	No	No
Kato^[13]^	2012	62/F	R	5 × 5 × 2	Regular	No	No
Kato	2012	45/M	L	6 × 3.5 × 2.5	Regular	No	No
Kato	2012	16/F	R	9.2 × 6 × 4	Regular	No	Yes
Kato	2012	54/F	L	11 × 6.5 × 3	Regular	No	No
Kato	2012	57/M	R	3 × 2 × 1.5	Lobular	No	No
Kato	2012	20/F	R	7 × 4 × 3	Regular	No	No
Kato	2012	57/M	L	3.0 × 2.5 × 2.0	Lobular	No	No
Kato	2012	12/F	L	9.5 × 6.5 × 5.0	Regular	No	No
			R	3.5 × 2.5 × 1.5	Regular	No	No
Kato	2012	36/F	R	8.2 × 6.4 × 3.0	Regular	No	No
Kato	2012	7/M	L	11.0 × 7.0 × 6.0	Lobular	No	No
Kato	2012	15/F	R	8.3 × 6.6 × 3.5	Lobular	Yes	Yes
Kato	2012	6/F	L	8.4 × 6.5 × 4.9	Regular	No	No
Kato	2012	62/F	L	10.7 × 4.1 × 2.9	Regular	No	No
Sánchez-Galán^[22]^	2014	3/F	N/A	N/A	N/A	N/A	N/A
Sánchez-Galán	2014	4/M	N/A	N/A	N/A	N/A	N/A
Sánchez-Galán	2014	4/F	N/A	N/A	N/A	N/A	N/A
Sánchez-Galán	2014	4/F	N/A	N/A	N/A	N/A	N/A
Sánchez-Galán	2014	5/F	N/A	N/A	N/A	N/A	N/A
Sánchez-Galán	2014	9/M	N/A	N/A	N/A	N/A	N/A
Sánchez-Galán	2014	11/F	N/A	N/A	N/A	N/A	N/A
Sánchez-Galán	2014	13/M	N/A	N/A	N/A	N/A	N/A
Huang^[23]^	2017	12/F	L	12 × 12 × 12	Regular	No	No
Jeon^[24]^	2017	6/M	R	4 × 3.5 × 2	Regular	No	No
Lambdin^[25]^	2018	42/F	L	23 × 10 × 10	Regular	No	No
Algazwi^[26]^	2020	18/F	R	N/A	Regular	No	No
Elnady^[27]^	2020	17/F	L	N/A	Regular	No	No
Brock^[14]^	2020	12/F	R	10 × 9.1 × 9.5	Regular	Yes	Yes
Aljuboori^[28]^	2021	30/NA	R	N/A	Regular	No	No
Tiwari^[29]^	2022	4/F	L	3.8 × 2.5 × 2.3	Regular	No	No
Nemoto^[30]^	2022	15/F	L	N/A	Regular	No	No
Chen^[31]^	2022	57/M	L	6.5 × 5.0 × 2	Regular	No	No
Current case	2023	15/F	R	14.5 × 12 × 5	Lobular	Yes	Yes

F = female, M = male, L = left, R = right, N/A = not available.

Among the thoracic GN cases, 6 (9.5%) were found to have caused bone erosion, including 3 cases of lobular GN and 3 of regular GN. All six patients were female, with a median age of 11 and an average age of 11. Additionally, 6 cases (9.7%) resulted in rib deformity. In the lobular thoracic GN cases, 3 (50%) caused bone erosion, while in the regular thoracic GN cases, 3 (4.6%) caused bone erosion. After statistical analysis (using R software version 4.2.2), we found a significant difference in the incidence of bone erosion between regular and lobular thoracic GN cases (Fisher’s exact test, *P* = .006 < .05, OR = .054, 95% CI: .0045–.56). Patients with lobular GN had a significantly higher probability of bone erosion than those with regular GN. This suggests that the appearance of lobular tumors may be related to bone erosion. Due to the small sample size, however, additional instances and research are required to confirm this link.

In the currently limited sample, the rare and aggressive growth pattern of GN that causes bone erosion was only found in female patients. This indicates that there may be a potential association between this destructive character of GN and patients’ gender. Of course, this also requires larger sample sizes and systematic analysis for verification. If these potential associations are validated, clinicians could receive more information to tailor personalized treatments for GN patients with different morphological features and genders. This could lead to better predictions of patient prognosis. For young female patients with lobular GN, GNs are more likely to be aggressive. Therefore, early surgery may be a more suitable treatment option to mitigate the potential risks of long-term follow-ups, such as the risk of the tumor continuing to grow and causing more damage.

Under CT, GN typically presents as a low-density mass with a round or elliptical shape (lower than the density of surrounding muscles), sometimes accompanied by punctate calcification. On enhanced scans, there is usually mild to the moderate enhancement and a delayed enhancement feature. On MRI, GN typically appears as a low signal on T1-weighted images and a high signal on T2-weighted images, sometimes with a “whorl” appearance. This appearance corresponds to the characteristic appearance of GN under a microscope where the interlacing of Schwann cells and collagen fibers is observed. ^[4,13,32,33]^ If GN has atypical CT and MRI Findings, the co-existence of malignant components should be considered.^[11]^ The CT and MRI findings, in this case, are consistent with previous reports, but imaging examination revealed that the tumor had an obvious lobular appearance and eroded adjacent bones. These conditions are extremely rare. Kato et al mentioned a case of lobular thoracic GN eroding the vertebral body and causing rib deformity in their study,^[13]^ but the erosion area, in that case, was relatively broad and superficial, while in this case, the erosion of the vertebral body was deeper and more severe. This suggests that in cases where GN erodes bones, their invasiveness may vary in different ways and degrees.

Thoracic GN needs to be differentiated from mediastinal masses such as thymoma, lymphoma, tuberculosis lymphadenitis, neurofibroma, schwannoma, and neuroblastoma. Although imaging examinations can help diagnose GN to a large extent, the final diagnosis still requires histopathological examination due to its low specificity. ^[34]^

Sánchez-Galán et al pointed out that the focus of treating GN is on personalized treatment plans. Complete removal of GN should only be considered as a treatment goal if it does not pose significant risks to the patient and surrounding structures. Imaging should be used to determine surgical risk factors and minimize the likelihood of surgical complications in GN surgical planning. It is important to note that incomplete removal may also be a reasonable choice due to the favorable prognosis of these tumors. In certain special cases, non-surgical treatment may be a reasonable option for treating GN.^[22]^ However, for GNs growing beside the thoracic vertebrae, short-term stability may lead clinicians to underestimate the potential damage caused by their long-term growth. Considering that thoracic GNs may cause problems such as scoliosis, spinal cord compression, bone erosion, and bone destruction during growth, we recommend adopting an early surgical resection strategy for thoracic GNs based on the individual characteristics of the patient to avoid the risks brought by long-term follow-up.

In terms of surgical options, thoracoscopy is a safe and efficient procedure for removing thoracic neurogenic tumors. It allows for enhanced visualization of the tumor and its connections and can facilitate a full excision without increasing the complexity or duration of the operation. Using this technique avoids the cosmetic and functional issues associated with a thoracotomy and results in a good cosmetic resection without tumor spillage,^[35]^ If a patient is diagnosed with intrathoracic GN and the tumor is small, with imaging evaluation showing no invasion of the spinal canal, thoracoscopy should be the preferred approach. If during surgery, the tumor is found to have tightly adhered to surrounding tissues or structures, or other factors make it difficult to complete the operation thoracoscopically, conversion to thoracotomy is also a reasonable option. If the tumor is giant, considering the good prognosis of GN with low recurrence rates,^[36,37]^ open surgery for segmental resection of the tumor is a good option, which can reduce damage to the paravertebral nerves and muscles. The use of different surgical methods can avoid damage to important blood vessels, nerves, and organs, and ensure complete exposure of the tumor. At the same time, the use of different surgical methods can reduce the surgical time, making it easier for patients to tolerate the procedure, and allowing the surgeon to conserve energy. This also improves the safety of the surgical process. Giant paraspinal GNs typically affect important organs such as the lungs, kidneys, intestines, and major blood vessels. Postoperative complications include cerebrospinal fluid leakage, paraplegia, intestinal obstruction, perioperative bleeding, pneumothorax, and others.^[38]^ When considering the resection of spinal ganglioneuromas, the surgical approach and extent of resection must be carefully weighed, while also considering the risks of surgical complications. Patients have a favorable long-term prognosis regardless of whether the tumor is excised or not.^[39]^

Currently, the only treatment for GN is surgical resection. However, Tao et al discovered that ganglioneuromas are driven by the activation of the AKT signaling pathway, which promotes cell growth and survival. The researchers also demonstrated that mTOR inhibitors, a class of drugs that target the mTOR pathway downstream of AKT, can effectively inhibit the growth of ganglioneuromas in zebrafish models. These findings suggest that mTOR inhibitors may represent a promising therapeutic strategy for the treatment of ganglioneuromas.^[40]^ If subsequent clinical trials can confirm that mTOR inhibitors can inhibit the growth of GN and reduce the volume of GN tumors in the human body, then targeted therapy to reduce tumor volume before surgery on large-volume GN, while reducing adhesion of the tumor to surrounding tissue, will greatly reduce surgical risks.

## 6. Conclusion

Our review of past literature suggests that a lobular appearance may be associated with a more aggressive growth pattern of thoracic GN, which may be more prevalent in young female patients. Therefore, young female patients with lobular GN may require more aggressive surgical strategies to avoid the risks associated with long-term follow-up. However, more cases and studies are needed to confirm and investigate these potential associations and the mechanisms behind them.

## Acknowledgments

Thank the Pathology Department of Ganzhou People’s Hospital for their professional help.

## Author contributions

**Data curation:** Haoxiang Zhuang.

**Formal analysis:** Zegang Ruan.

**Supervision:** Chenyang Xu.

**Writing – original draft:** Haoxiang Zhuang, Zegang Ruan.

**Writing – review & editing:** Chenyang Xu.
